# Evaluation of the Bioactivities of *Rumex crispus* L. Leaves and Root Extracts Using Toxicity, Antimicrobial, and Antiparasitic Assays

**DOI:** 10.1155/2019/6825297

**Published:** 2019-10-30

**Authors:** Oladayo Amed Idris, Olubunmi Abosede Wintola, Anthony Jide Afolayan

**Affiliations:** Medicinal Plants and Economic Development (MPED) Research Centre, Department of Botany, University of Fort Hare, Alice 5700, South Africa

## Abstract

Traditional folks in different parts of the world use *Rumex crispus* L. for the treatment of microbial infections, malaria, and sleeping sickness in the form of decoction or tincture. In the search for a natural alternative remedy, this study aimed to evaluate the antimicrobial, antitrypanosomal, and antiplasmodial efficacy and the toxicity of *R. crispus* extracts. Antimicrobial potency of the extracts was evaluated using the agar dilution method to determine the minimum inhibitory concentration (MIC). The antitrypanosomal activity of the extracts was evaluated with the *Trypanosoma brucei brucei* model while the antimalaria potency was tested using *Plasmodium falciparum* 3D7 strain. Toxicity was then tested with brine shrimp assay and cytotoxicity (HeLa cells). The acetone extract of the root (RT-ACE) reveals the highest antimicrobial potency with the lowest MIC value of <1.562 mg/mL for all bacteria strains and also showed high potent against fungi. RT-ACE (IC_50_: 13 *μ*g/mL) and methanol extract of the leaf (LF-MEE; IC_50_: 15 *μ*g/mL) show a strong inhibition of *P. falciparum.* The ethanol extract of the root (RT-ETE: IC_50_: 9.7 *μ*g/mL) reveals the highest inhibition of *T.b. brucei* parasite. RT-ETE and RT-ACE were found to have the highest toxicity in brine shrimp lethality assay (BSLA) and cytotoxicity which correlates in the two assays. This research revealed *Rumex crispus* has potency against microorganisms, *Trypanosoma*, and *Plasmodium* and could be a potential source for the treatment of these diseases.

## 1. Introduction

Medicinal plants have been used for ages in several ways for antimicrobial, hypoglycemic, antihelminthic, antiulcerogenic, hepatoprotective, antipyretic, analgesic, insecticidal, piscicide, pesticide, pharmaceutical, and cosmetic purposes [1]. The biological activities and efficacy of medicinal plants have been tested in traditional medicine and in several fields of science [2]. Their potency and curative potentials were documented independently across the globe suggesting that plants could be a potential source of novel drugs [2–4]. There has been a rapid increase in the use of medicinal plants as a curative prototype, and new products are emanating from these natural products into the commercial market daily. These products are believed by the consumers to be safer and effective than the chemotherapeutic conventional drugs [4, 5]. It was estimated that about 61% of newly developed drugs between 1981 and 2002 have their source from plants and are successful in the treatment of cancer and several other diseases [2, 6]. Many medicinal plants have been screened for antimicrobial, antiplasmodial, and antitrypanosomal activities, and some of these plants showed prospect in the treatment of these infections [3, 7, 8]. In most cases, the medicinal properties of a plant are a function of its phytochemical constituents [9].

### 1.1. Resistance of Microorganisms to Drugs

There have been reports of multiple drug resistance (MDR) in the human pathogenic microbial organisms due to indiscriminate use of commercially available drugs [4]. The capacity of diseases to drug resistance is the clinical determinants of the pathogenesis of a particular pathogen. For instance, there have been reports of higher resistance to a group of antibiotics in an area where it is regularly misused [10, 11]. Multiple drug-resistant strains of pathogens and parasites are economically important, and these pose a huge threat to the public health system. This calls for an immediate intervention with new drugs that use a different mechanism of action in the treatment of these diseases. On the other hand, there are several scientific evidences to the success of medicinal plants in treating diseases; hence, plants could be a novel source that will combat drug resistance [5].

Plants are known to produce complex bioactive organic compounds that possess several bioactivities. The compounds are secreted in various parts of the plants such as seeds, fruits, flowers, leaves, roots, stems, bark and some plant secretions (isothiocyanates, plant pigment, plant gum, phytoalexins, essential oils, and hydrolytic enzymes) [12]. The bioactive constituent in medicinal plants has shown tremendous health benefits. For instance, the major antibacterial bioactive compounds in plants are anthraquinones and flavonoids which are active against several human pathogenic bacteria [13]. Phenolic compounds, flavonoids, and artemisinin are active against malaria causing parasite, *Plasmodium falciparum* [14]. It was also reported that flavonoids and glycosides in plants inhibit the growth of African sleeping sickness (*Trypanosoma brucei*) parasites [15]. The increase in the occurrence and sustenance of drug-resistant pathogens to drugs has drawn the attention of scientists and pharmaceutical communities towards harnessing the potentials of plant-derived substances used successfully in different countries by traditional medical practitioners [16].

### 1.2. Tests for Toxicity, Antimicrobial, and Antiparasite

There is a rapid increase in interest and demand for medicinal plants in various parts of the world because they are considered natural, safer, and cheaper [17]. On the other hand, there is a general concern in the scientific communities on the safety, efficacy, and pharmacokinetics of medicinal plants, irrespective of their wide acceptance and usage. The possible side effects of plant products cannot be ruled out regardless of their adoption as a novel source of drugs [18, 19].

The source of the toxicity of plants may be due to the contaminants in the environment or from the plant-secreted phytochemicals [20, 21]. Different biological models have been tested via *in vitro* and *in vivo* assays to qualify and quantify the potential toxicity of extracts of medicinal plants. However, recent studies employed the use of preliminary toxicity assessment tools using brine shrimp species: *Artemia franciscana*, *Artemia salina*, *Thamnocephalus platyurus*, and *Artemia urmiana* [22–24], which is considered a useful tool to evaluate the potential toxicity of plant extracts. Cytotoxic screening using HeLa human cervix carcinoma cells is an advanced tool to screen substances inducing apoptosis. In addition, a comparative toxicity study of plants extract could be tested using *in vivo* study. The result, however, showed quite a good correlation in the mean value of *in vitro* study lethal concentration (LC_50_) and the acute oral lethal dose (LD_50_) of *in vivo* study [23, 25].

*Rumex crispus* L. (curled dock) is commonly used by traditional healers for treatment of various diseases and correction of disorders such as gastrointestinal tract disorders, antihelminthic diseases, anti-inflammatory, and arthritis, and it is also used as laxative, antipyretic, antioxidant, and antimicrobial [5, 26]. More so, there are reports of the antimicrobial [27–29], antitrypanosomal [30], and antiplasmodial [31] properties and toxicity of *R. crispus* which were evaluated in this research. This is necessary to validate the claim of traditional healer practitioners in Eastern Cape, South Africa, and any other part of the world that use *R. crispus* for the treatment of these ailments.

## 2. Materials and Methods

### 2.1. Organic Extraction

The leaves and roots of *Rumex crispus* were collected from a fallow damp field, University of Fort Hare (32°47′1.23″S, 26°51′9.85″E), in the summer of 2017. It was identified, and a sample of the specimen was placed in Giffen herbarium (Idr-Med-2017/03), University of Fort Hare. The leaves and roots of the plant specimen were carefully sorted, and the infected ones were removed. The specimen was rinsed, dried in an oven at 30°C until a fixed weight, and then pulverized with a mechanical grinder (Polymix PX-MFC90D, Switzerland). The pulverized sample was stored in an airtight container, in a refrigerator at 4°C until use for extraction.

Crude extraction of *R. crispus* was done using standard procedures. The choice of solvent for the extraction process is an imperative factor that must be considered during the antimicrobial and toxicity tests. The extracting potentials of different solvents differ; therefore, extraction was done using solvents of low reactivity: a technically graded acetone, methanol, ethanol, and distilled water. The extractant (solvent) and the plant materials used were in the ratio of 1 :10 (g/mL). The dried root (RT) and the aerial part (LF) of the plant sample (100 g) were macerated with the extractant solvents (1000 mL) at room temperature. The crude extract of methanol (MEE), ethanol (ETE), and acetone (ACE) were concentrated under reduced pressure by using a rotatory evaporator, considering the boiling point of the respective solvent, and then dried in a desiccator at a mild temperature (40°C). The filtrate of water samples (WAE) was chilled at −40°C with a shell refrigerant (PolyScience AD15R-40-A12E, USA) and freeze-dried (Savant vapour trap, RV-T41404, USA) for 48 h. The plant crude extracts were stored in sterile bottles and refrigerated at 2 to 4°C until further use.

### 2.2. Determination of Minimum Inhibitory Concentrations (MICs) of the Extracts

Minimum inhibitory concentrations (MICs) were used to determine the lowest concentration of the extracts that will inhibit visible microbial growth after an incubation period of 24 h (bacteria) and 48 h (fungi). Usually, MICs are diagnostic laboratory tools used to confirm microbial resistance but are often used in research laboratories to determine the *in vitro* activity of antimicrobials and its MIC breakpoints [32].

### 2.3. Antibacterial Activity of the Plant Extracts

The potency of each extract of *R. crispus* was tested against four strains of Gram-negative (*Klebsiella pneumonia* ATCC 4352, *Pseudomonas aeruginosa* ATCC 19582, *Escherichia coli* ATCC 8739, and *Vibrio cholerae* ATCC 14033) bacteria and four strains of Gram-positive (*Bacillus subtilis* ATCC 6633, *Staphylococcus aureus* ATCC 43300, *Streptococcus pyogenes* ATCC 19615, and *Bacillus cereus* ATCC 33019) bacteria. These strains of bacteria were collected from the Department of Microbiology, University of Fort Hare, South Africa. The concentration of the plant extracts that inhibits the microbial growth after 24 h of incubation was determined by MIC assay as described [33] with modification. The agar dilution method was used for the MIC to determine the antibacterial efficiency of *R. crispus*, and the resultant efficacy was compared with a commercial antibiotic drug (erythromycin).

The collected strains of bacteria were subcultured for 24 h at 37°C in a sterilized Mueller–Hinton Agar (Oxoid™), following the manufacturer preparation instructions. The bacteria were harvested using 5 mL of sterilized saline solution, and the inoculum was adjusted to 10^6^ CFU/mL by comparison with 0.5 McFarland standard {0.5 mL of 0.048 M BaCl_2_ (1.17% w/v BaCl_2_·2H_2_O) + 99.5 mL of 0.18 M H_2_SO_4_ (1% v/v)} between 106 and 108 CFU/mL and then the inoculum was applied to the agar prepared in sterilized Petri plates [13]. A dilution range of 1.562–25 mg/mL was prepared for the plant extracts, and concentration range of 0.5–8 *μ*g/mL of erythromycin was used for the positive control. The plant's extracts were dissolved in 10% dimethylsulphoxide (DMSO) (except aqueous extracts) and distilled water and then purified by filtering through Millipore filter paper. Mueller–Hinton agar was prepared following the manufacturer's instructions; a volume of 20 mL of the agar was measured in Erlenmeyer flask and autoclaved. Thereafter, 1 mL of the prepared extract was added, swirled, and poured into sterile disposable Petri dishes. The agar was allowed to solidify at room temperature. The standardized inoculum was diluted in sterile saline solution and Mueller–Hinton broth in ratio 1 : 10, to attain 0.5 McFarland standard between 106 and 108 CFU/mL. An aliquot volume of 0.1 mL of the inoculum was pipetted and transferred to the agar medium, and the plates were incubated for 24 h at 37°C.

### 2.4. Antifungal Screening

The fungi *Trichophyton tonsurans* ATCC 28942, *Trichosporon mucoides* ATCC 201382, *Penicillium aurantiogriseum* ATCC 16025, *Penicillium chrysogenum* ATCC 10106, *Candida glabrata* ATCC 66032, and *Candida albicans* ATCC 32077 were collected from the Department of Botany, University of Fort Hare, and subcultured on Sabouraud Dextrose Agar (Oxoid™), at 30°C for 48 h. The antifungal assay was carried out according to the method described by Lass-Flörl et al. [34], with modification. The medium (Sabouraud Dextrose Agar) was prepared following the manufacturer's instructions, and 5 mg/100 mL of chloramphenicol was added to the agar media before autoclaving to inhibit bacteria growth. The suspensions of the inoculum were transferred into a sterile saline solution from the subculture, by carefully collecting the conidia with a sterile cotton swab. The suspension was homogenized for 15 s with a vortex (2,000 rpm), and the inoculum was diluted in Mueller–Hinton broth in ratio 1 : 10, to obtain a final test inoculum load range between 1.0 × 10^6^ and 5.0 × 10^6^ CFU/mL. The adjusted working inoculum was plated on solidified sterilized Sabouraud Dextrose Agar plates with plant extract's dilution range of 0.625–10 mg/mL, and the control agar plates was diluted at the range of 1–16 *μ*g/mL of nystatin; all plates were incubated for 48 h at 30°C.

### 2.5. Screening of *R. crispus* against *Plasmodium falciparum*

The malaria parasites (*Plasmodium falciparum*, sensitive strain; 3D7) were used in blood-stage culture to test the antimalarial efficacy of *R. crispus* extracts through *in vitro* studies, following the method described by Murugan et al. [7]. The parasites were preserved in RPMI 1640 medium containing 2 mML glutamine and 25 mM Hepes (Lonza). The medium was further supplemented with 5% Albumax II, 20 mM glucose, 0.65 mM hypoxanthine, 60 *μ*g/mL gentamycin, and 2–4% hematocrite human red blood cells. The parasites were cultured at 37°C under an atmosphere condition of 5% CO_2_, 5% O_2_, and 90% N_2_ in sealed T75 culture flasks. The infected erythrocytes were transferred into a fresh medium to propagate the culture, and parasitaemia (the concentration of parasites in the culture) was measured by light microscopy of Giemsa-stained thin blood smears.

In the experiment, the tested extracts were prepared in 0.4% dimethyl sulfoxide (DMSO). Then, the parasiticidal potency of tested extracts was done by single concentration screening. The single concentration screening was conducted by adding 25 *μ*g/mL (extracts) to parasite cultures in 96-well, clear plates and incubated for 48 hours in a CO_2_ incubator at 37°C. After 48 hours, 20 *μ*L of culture was removed from each well and combined with 125 *μ*L of a mixture of Malstat and NBT/PES solutions in a fresh 96-well plate. These solutions were used to measure the activity of the parasite lactate dehydrogenase (pLDH) enzyme in the cultures. A purple product is formed when pLDH is present [8], and this product is quantified in a Spectramax M3 microplate reader at the absorbance of 620 nm. For each extract concentration, percentage parasite viability and pLDH activity in extract-treated wells relative to untreated controls were calculated. The extracts were tested in triplicates, and standard deviations (SD) were derived. For comparative purposes, chloroquine (a standard antimalarial drug) was used as a standard (IC_50_ values ranging from 0.01 to 0.05 *μ*M).

### 2.6. Antitrypanosomal Activity of *R. crispus* Extracts

The subspecies responsible for sleeping sickness in Nagana, *Trypanosoma brucei brucei* (*T.b. brucei*), is infective to humans and is commonly used as a drug screening model in the laboratory. This research adopted the *T.b. brucei* model as described in [35], with modification. The blood-stage forms of *T.b. brucei* (Strain 427) were grown in Iscove's Modified Dulbecco's Medium (IMDM) enhanced with 10% fetal bovine serum, and the culture was incubated in 5% CO_2_, at 37°C.

For primary screening of extracts' trypanocidal activity, a single concentration of 50 *μ*g/mL in duplicate was prepared from the extract stock in IMDM and added to the culture of *T.b. brucei* in a 96-well plate. The mixture was incubated for 48 h, and the number of survived parasites was determined by adding a resazurin-based reagent which would be reduced to resorufin by living cells. Resorufin is a fluorescent compound (Excitation560/Emission590); hence, absorbance was measured in a multiwell fluorescence plate reader (Spectramax M3) to determine viable cells. The percentage parasite viability of each extract was determined by comparing the resorufin fluorescence in treated wells in relation to untreated controls. The resultant was then plotted against log (extract concentration), and the IC_50_ from the resulting dose response in nonlinear regression was obtained. The percentage parasite viability and response to extracts were then compared with pentamidine (a drug for trypanosomiasis) as a positive control.

### 2.7. Toxicity Test

The toxicity of plant was tested by exposing the eggs and larvae of *Artemia salina* to the plant extracts and comparing their LC_50_ values with toxic compound (potassium dichromate: K_2_CrO_7_), suitable as a positive control for this test. Further toxicity was done by testing extracts on HeLa cells (human cervix adenocarcinoma). All experiments were carried out in accordance with the guidelines for the Care and Use of Laboratory Animals. The research methodology was approved by the University of Fort Hare, Animal Ethical Committee (reference number: AFO121SIDR01).

#### 2.7.1. Brine Shrimp Hatchability Assay (BSHA)

**Figure 1:**
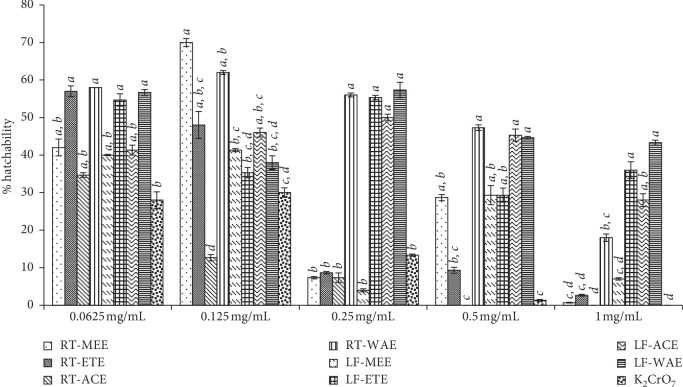
Percentage hatchability of *A. salina* cysts incubated for 72 h in different solvent extracts and five concentrations. The values are means of hatchability potential for *R. crispus* extracts/control ± SD of three replicates. The bars within a concentration with different letters are significantly different; mean values of *a* > *b* > *c* > *d*. RT-MEE: methanol extract of root; RT-ETE: ethanol extract of root, RT-ACE: acetone extract of root, RE-WAE: water extract of root, LF-MEE; methanol extract of leaf, LF-ETE: ethanol extract of leaf, LF-ACE: acetone extract of leaf and LF-WAE: water extract of leaf.

The BSHA was carried out as previously described by Otunola et al. [36], with modification. The eggs of *Artemia salina* (brine shrimp) were obtained from aquaculture outlet in East London, South Africa. The eggs of *Artemia salina* was hatched at optimum condition, in seawater (salinity 38 ppt and pH 8.5), collected in East London, South Africa (32° 59 S 27°52 E). The concentration of the extracts and a standard drug (K_2_CrO_7_) were graded (0.0625, 0.125, 0.25, 0.5, and 1.0 mg/mL) by serial dilution in 30 mL of filtered seawater, in sterile Petri dishes. Each concentration of extracts and positive and negative control were tested in triplicate. Ten eggs of brine shrimp were introduced into the Petri dishes. The hatched nauplii were counted against the ratio of the number of eggs introduced at the beginning of the experiment at 12 h interval, and the level of toxicity was evaluated using probit regression (Figure 1).

#### 2.7.2. Brine Shrimp Lethality Assay (BSLA)

**Figure 2:**
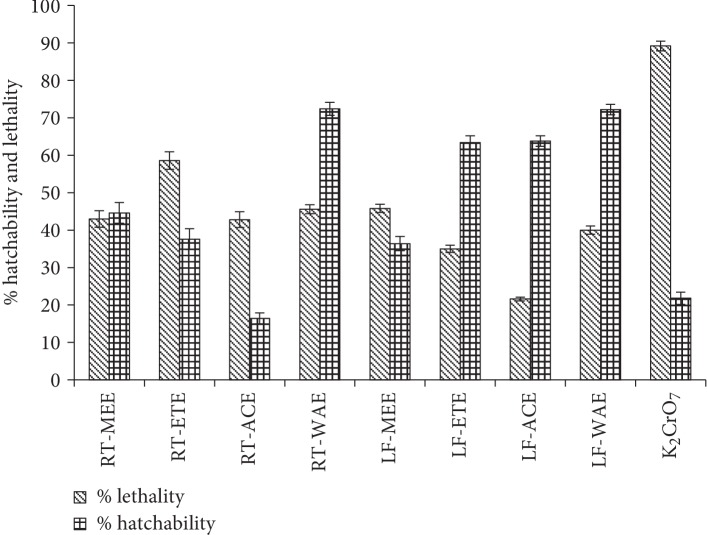
Overall percentage hatchability and lethality of the extracts of *R. crispus* and the standard K_2_CrO_7_ after 72 h incubation period. The values are means of hatchability and lethality potential for all the concentrations of the extracts/control ± SD of replicates. RT-MEE: methanol extract of root; RT-ETE: ethanol extract of root; RT-ACE: acetone extract of root; RE-WAE: water extract of root, LF-MEE: methanol extract of leaf; LF-ETE: ethanol extract of leaf; LF-ACE: acetone extract of leaf; and LF-WAE: water extract of leaf.

**Figure 3:**
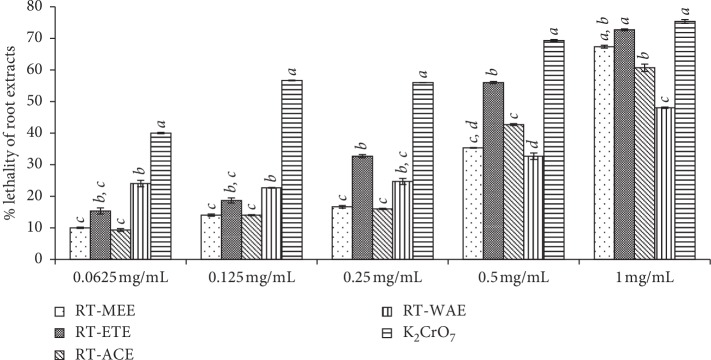
Percentage mortality of *Artemia salina* larva in different concentrations of the root extracts of *R. crispus* and control, in a test period of 72 h. The values are mean of the triplicates of mortality in each plant extracts (mean ± SD). The bars within a concentration, with different letters, are significantly different; mean values of *a* > *b* > *c* > *d*. RT-MEE: methanol extract of root; RT-ETE: ethanol extract of root; RT-ACE: acetone extract of root; RE-WAE: water extract of root; LF-MEE: methanol extract of leaf; LF-ETE: ethanol extract of leaf; LF-ACE: acetone extract of leaf; LF-WAE: water extract of leaf.

**Figure 4:**
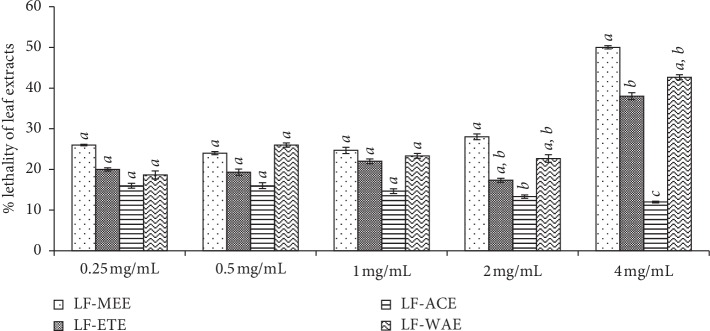
Percentage mortality of *Artemia salina* larva in different concentrations of the leaf extracts of *R. crispus*, in a test period of 72 h; The values are mean of the triplicates of mortality in each plant extracts (mean ± SD). The bars within a concentration with different letters are significantly different; mean values of *a* > *b* > *c* > *d*. RT-MEE: methanol extract of root; RT-ETE: ethanol extract of root; RT-ACE: acetone extract of root; RE-WAE: water extract of root; LF-MEE: methanol extract of leaf; LF-ETE: ethanol extract of leaf; LF-ACE: acetone extract of leaf; LF-WAE: water extract of leaf.

In the study, BSLA was carried out adopting the method as described in [37], with slight modifications. The brine shrimp eggs were incubated at the optimum condition in a glass jar for 48 h. A count of 10 nauplii with an inverted microscope was pipetted into different Petri dishes with a graded concentration of extracts, potassium dichromate, and control seawater. The lethality of larvae was observed with an inverted microscope at an interval of 12 h, and the total lethality was computed using the Standardized Lethality Index (SLI). The condition of the exposed larvae was evaluated with SLI, as used by Agrafioti et al. [38]. Based on the index, each experimental nauplius was ranked from 0 to 3, to measure the intensity of the response. In the scale; responses 0: normal nauplii, responses 1: nauplii were knocked down but were able to swim for short intervals, responses 2: nauplii were knocked down and unable to swim but with visible movement, and responses 3: nauplii were dead (Table 1, Figures 2–4). Thus, the lethality index was calculated with the equation:

(1) $$\begin{array}{c} \text{lethality index}=\overset{4}{\underset{n=0}{\sum }}\left[\frac{\left(\;N_{0}\times W_{1}\right)+\left(N_{1}\times W_{2}\right)+\left(N_{2}\times W_{3}\right)+\left(N_{3}\times W_{4}\right)}{N_{\text{t}}\times N_{\text{ob}}\times {\text{Max}}_{w}}\right]\times 100, \end{array}$$

where *N*_0_: number of nauplii at rank 0; *N*_1_: number of nauplii at rank 1; *N*_2_: number of nauplii at rank 2; *N*_3_: number of nauplii at rank 3; *N*_t_: total number of nauplii; *N*_ob_: number of observations (three days, six observations); Max_*w*_: maximum lethality coefficient; and lethality coefficients: *W*_1_: 0.0, *W*_2_: 0.17, *W*_3_: 0.33, *W*_4_: 0.50, *W*_*i*_=*i* /(0 +1 +2 +3 ), *i*=0,1,2,3.

#### 2.7.3. Cytotoxicity Assay

The cytotoxicity of the extracts of *R. crispus* was estimated using HeLa (human cervix adenocarcinoma) cells as described by Larayetan et al. [8]. The stock solutions were prepared in 1% DMSO to attain a final concentration of 50 *μ*g/mL. The HeLa cells were incubated at a fixed concentration (50 *μ*g/ml) of the extracts in 96-well plates for 48 h. The viability of the cells after treatment with extracts was determined by adding resazurin-based reagent and reading resorufin fluorescence in a microplate reader (Spectramax M3). The results were expressed as percentage viability in resorufin fluorescence-treated extracts per wells relative to the untreated wells (controls). The cytotoxicity of the extracts was done in duplicate, and the positive control, standard drug (emetine), was used for comparative reference.

### 2.8. Statistical Analysis

The mean of the triplicates of the results for the assay was expressed as mean ± SD. The data were analyzed statistically by one-way analysis of variance, and differences between data were extrapolated by Fisher's Least Significant Difference (LSD) test, where it is applicable. The relationship between the response to the stimulus of the independent variables (concentration) and the quantal (dead or alive, all or none) was analyzed by probit regression to estimate the LC_50_ and LC_99_ using statistical software (SPSS).

## 3. Results

### 3.1. Antimicrobial Activity of Plant Extracts

The antibacterial and antifungal potency of the extracts is given in Tables 2 and 3. The result shows that all extracts of *R. crispus* have a varying degree of antimicrobial activities with a variation in the level of potency.

#### 3.1.1. Minimal Inhibitory Concentration (MIC) of Plant Extracts

The bactericidal properties of *R. crispus* was conducted using a graded concentration of the plant extracts, to find the minimal inhibitory concentration (MIC) of the extracts against predispose bacterial strains (Gram-negative: *K. pneumoniae*, *P. aeruginosa*, *E. coli*, and *V. cholera*; Gram-positive: *B. subtilis*, *S. aureus*, *S. pyogenes*, and *B. cereus*). The concentration effect of the plant extracts and standard drug is reported in Table 2. The MIC was confirmed by the absence of the growth of tested bacterial strains on the surface of the agar which shows the potential bactericidal activity of the plant's extracts against the tested bacteria.

The acetone extract of the root (RT-ACE) registered the highest antimicrobial potency with the lowest MIC value of <1.562 mg/mL, in all the experimental strains of bacteria. Also, the ethanol extract of the root (RT-ETE) and methanol extract of the root (RT-MEE) show high inhibitory capacity of the bacteria. On the contrary, the MIC of water extract of the root (RT-WAE), water extract of the leaf (LF-WAE), and acetone extract of the leaf (LF-ACE) was low. The methanol extract of the leaf (LF-MEE) and ethanol extract of the leaf (LF-ETE) showed a moderate but an undulating MIC on the bacterial strains. The mean MIC value of *R. crispus* root extracts proved to have significant effects against bacterial strains, more than the leaf extracts, which aligns with previous research as reported by Mostafa et al. [33], on how extracts of the underground parts of many plants could be more active than the aerial parts.

#### 3.1.2. Antifungal Activity of Plant Extracts

Antifungal susceptibility testing remains less developed compared to antibacterial testing. However, it is an adoption of antibacterial testing. The knowledge of the mechanism of fungal resistance to drugs through the MIC method has been valuable in identifying and measuring resistance in *in vitro* systems [34]. In the study, the endpoint was read visually with a viewing mirror and the growth level (MIC) was recorded (Table 3). The highest level of MDR was recorded in *C. glabrata* for all the extracts (>10 mg/mL) as recorded in Table 3. High resistance was also recorded in *P. chrysogenum* and *C. albicans* across the extracts and the positive control. It was established by Lass-Flörl et al. [34] that some fungi (mold) are considered resistant to some antifungal drugs (itraconazole) at MICs ≥8 *μ*g/mL but susceptible to MICs of 0.125–0.5 *μ*g/ml. The MICs of the extracts against tested fungal extracts showed varying concentrations as shown in Table 3. The overall MIC of leaf extracts showed a higher antifungal potency, contrary to the lesser MIC of leaf in the antibacterial test. This may be due to the slight differences in the phytochemical composition of the leaf and root of *R. crispus*.

### 3.2. Antiplasmodial Activity of *R. crispus* Extracts

**Figure 5:**
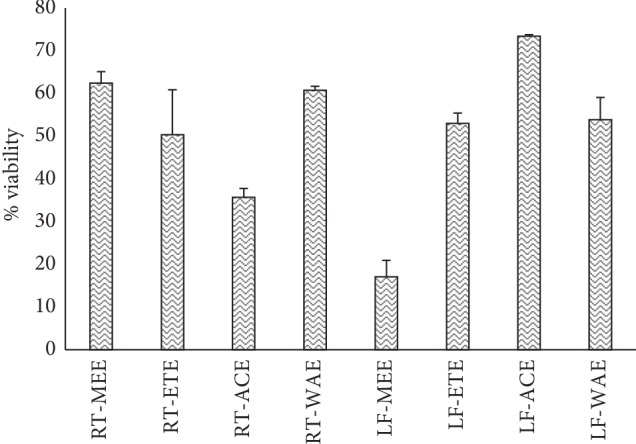
Bar graph shows the percentage parasite (*P. falciparum*) viability ± SD tested against *R. crispus* extracts. RT-MEE: methanol extract of root; RT-ETE: ethanol extract of root; RT-ACE: acetone extract of root; RE-WAE: water extract of root; LF-MEE; methanol extract of leaf; LF-ETE: ethanol extract of leaf; LF-ACE: acetone extract of leaf; LF-WAE; water extract of leaf.

**Figure 6:**
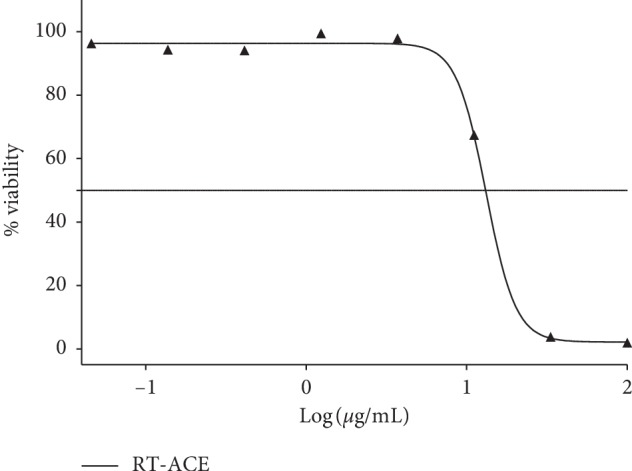
Dose-response plots and % viability of parasite to RT-ACE (acetone extract of the root). IC_50_: 13 *μ*g/mL.

**Figure 7:**
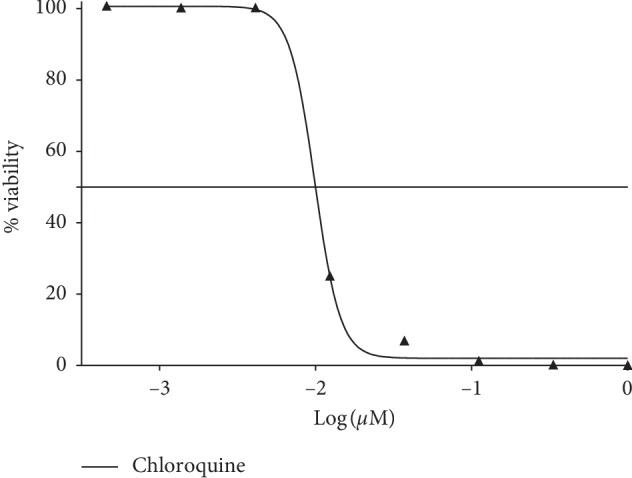
Dose-response plots and % viability of parasite to chloroquine standard. IC_50_: 10 *μ*M.

The solvent extracts of *R. crispus* were subjected to an *in vitro* screening of malaria-causing parasite (*P. falciparum* strain 3D7). The percentage viability of the parasite is equivalent to plasmodium lactate dehydrogenase (pLDH) enzyme released. The extracts RT-ACE (35.72 ± 2.08%) and LF-MEE (17.11 ± 3.82%) caused a significant decrease in the level of pLDH, causing viability of the parasite to be less than 50% (Figure 5). Due to the bioactive of RT-ACE and LF-MEE, they were put forward for pLDH IC_50_ (50% inhibitory concentration) screening and compared with the positive control (chloroquine). Their percentage viabilities were then plotted against the logarithm of extract concentration (250 to 0.11 *μ*g/mL), a 3-fold dilution, but chloroquine was in the concentration of 0.01–0.05 *μ*M. The IC_50_ values were obtained from the resulting dose-response curve by nonlinear regression; IC_50_ values of RT-ACE, LF-MEE, and chloroquine were evaluated: 13 *μ*g/mL, 15 *μ*g/mL, and 10 *μ*M, respectively, as shown in Figures 6 and 7.

### 3.3. Antitrypanosomal Activity of *R. crispus* Extracts

**Figure 8:**
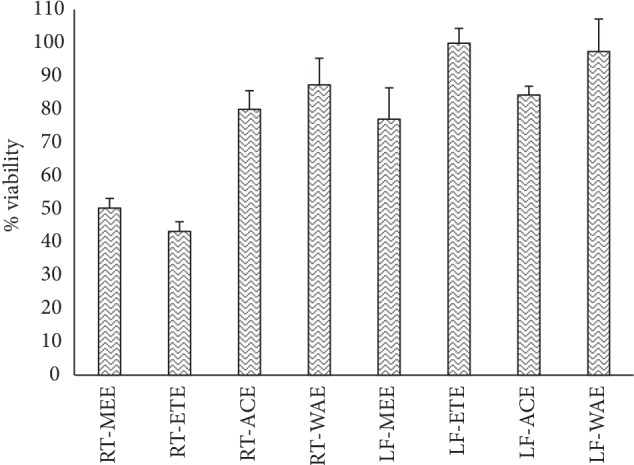
Percentage viability of *T.b. brucei* obtained from the test against extracts of *R. crispus*. RT-MEE: methanol extract of root; RT-ETE: ethanol extract of root; RT-ACE: acetone extract of root; RE-WAE: water extract of root; LF-MEE: methanol extract of leaf; LF-ETE: ethanol extract of leaf; LF-ACE: acetone extract of leaf; and LF-WAE: water extract of leaf.

**Figure 9:**
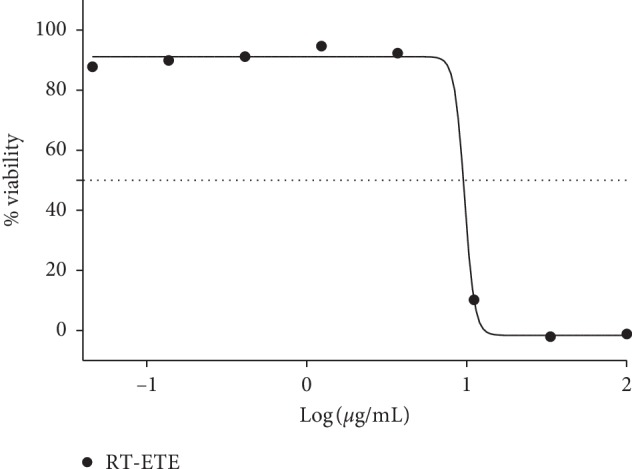
Percentage viability and dose-response curve of *T.b. brucei* to RT-ETE (ethanol extract of root). IC_50_: 9.7 *μ*g/mL.

**Figure 10:**
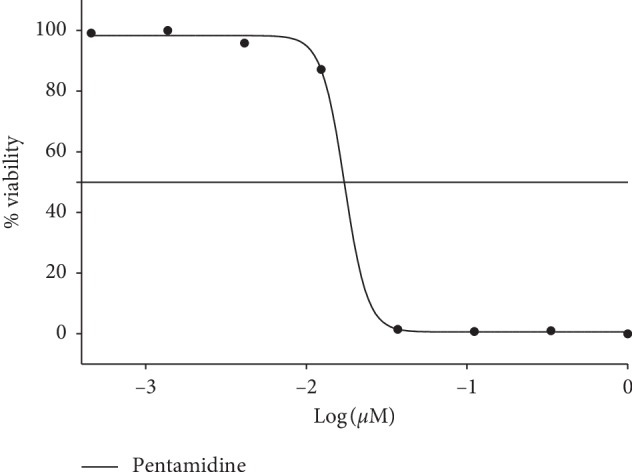
Percentage viability and dose-response curve of *T.b. brucei* to pentamidine (standard control). IC_50_: 0.017 *μ*M.

The potential of *R. crispus* extracts was tested on *T.b. brucei* parasite, causative agents of African sleeping sickness. The ethanol extract of the root of *R. crispus* (RT-ETE) has the highest potency among the extracts, acting against the parasite and reducing the viability to 43.27 ± 2.92% as shown in Figure 8. RT-ETE was thereafter put forward for further IC_50_ screening, and the outcome was compared to the IC_50_ of pentamidine (standard control): IC_50_: 9.7 *μ*g/mL and IC_50_: 0.017 *μ*M, respectively. The IC_50_ was obtained from the dose-response curve extrapolated by nonlinear regression using percentage viabilities plotted against logarithm (Figures 9 and 10).

### 3.4. Toxicity Test

#### 3.4.1. Brine Shrimp Hatchability

The percentage inhibition of the hatchability of the eggs of *Artemia salina* after 72 h exposure to the plant extracts and standard drug at optimum incubation conditions is shown in Figure 1. The entire tested extract composite shows some degree of inhibition of the rate of hatching of *A. salina* eggs compared to the control. The highest percentage of successfully hatched eggs into larvae in the least concentration used (0.0625 mg/mL) was observed in the extracts RT-ETE (57%), RT-WAE (58%), LF-ETE (54.67%), and LF-WAE (56.67%). This could be due to the polarity of the extraction solvents (ethanol and water), which brings about the fair inhibition of hatchability of the brine shrimp eggs. The hatching success of the cysts in RT-ACE has no significant difference, compared to the positive control (K_2_CrO_7_). At the concentration 0.125 mg/mL of the tested samples, there was an overall more successful hatch of the cyst with the highest percentage in the extract of RT-MEE (70%), while the least was in RT-ACE (12.67%). At the highest concentration of 1 mg/mL, the toxicity of the extracts expressed their potency as postulated. The extracts of the root of *R. crispus* showed higher toxicity compared to the leaf extracts, and hence, the success of hatchability was very low (Figure 1). The overall percentage hatchability of *A. salina* cyst against the extracts is shown in Figure 2. The water extracts of the root (72.4%) and leaf (72.2%) are not significantly different; they have the highest hatchability and hence less toxic compared to other extracts. The acetone extract of the root (RT-ACE) has the lowest percentage hatchability, and it is not significantly different from the standard drug (K_2_CrO_7_) with an inhibition percentage of 16.4% and 21.8%, respectively (Figure 2).

#### 3.4.2. Brine Shrimp Lethality

The exposure of *A. salina* to toxins could have led to an elevation of glutathione S-transferase (sGST) activity in the phase II detoxication system of the larva. The depletion of glutathione levels and intracellular thiols, which act against oxidative stress in this organism, by these toxins leads to the death of the larva [39]. In most cases, the difference between toxins and nontoxic is dose or concentration. The lethal concentration (LC_50_ and LC_99_) of the extracts and the standard toxic drug was evaluated with probit regression and recorded as shown in Table 1. In the study, the level of toxicity of the extracts and the control in descending order is as follows: K_2_CrO_7_ (LC_50_ 0.101 mg/mL), RT-ETE (LC_50_ 0.275 mg/mL), RT-ACE (LC_50_ 0.363 mg/mL), RT-MEE (LC_50_ 0.491 mg/mL), RT-WAE (LC_50_ 0.612 mg/mL), LF-ACE (LC_50_ 1.824 mg/mL), LF-MEE (LC_50_ 2.734 mg/mL), LF-WAE (LC_50_ 2.990 mg/mL), and LF-ETE (LC_50_ 4.658 mg/mL) (Table 1). The overall percentage mortality of all concentrations of the extracts of *R. crispus* in 72 h, as shown in Figure 6, indicated that there was no significant difference between the toxicity of the extracts of RT-MEE (43%), RT-ACE (42.8%), RT-WAE (45.6%), and LF-MEE (45.8%). The highest toxicity among the extracts was RT-ETE (58.6%), while K_2_CrO_7_ has the mortality of 89.2% as the control. The acetone extract of the leaf (LF-ACE) has the lowest mortality of 21.6% among all tested samples contrary to the acetone extract of the root (RT-ACE).

**Figure 11:**
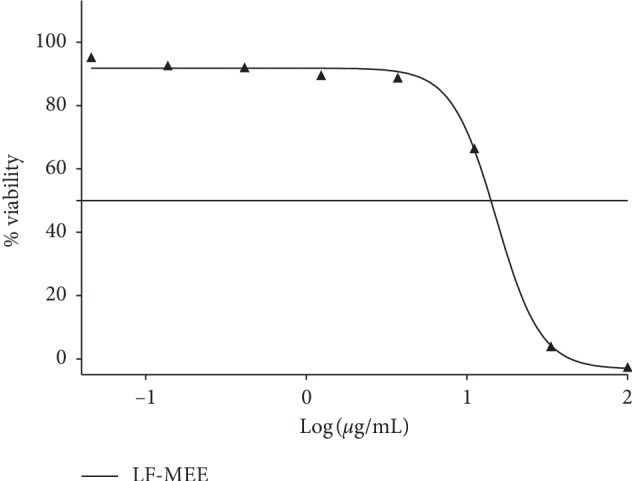
Dose-response plots and % viability of parasite to LF-MEE (methanol extract of the leaf). IC_50_: 15 *μ*g/mL.

The percentage sensitivity of the larva to extracts per each concentration used was tested and the effect of the various concentrations of the plant extracts used on the mortality of the larvae is shown in Figures 3 and 4. At the lowest concentration (0.0625 mg/mL) of the root extracts followed by 0.125 mg/mL, the toxicity of the extracts and the control drug was not fully expressed. The percentage lethality of all the extracts at these concentrations (0.0625 mg/mL and 0.125 mg/mL) as shown in Figure 3 was low and less than 50% of the shrimp were knocked down. At the concentration 1 mg/mL, there is no significant difference (*P* < 0.05) between the toxicity of RT-MEE (67.3%), RT-ETE (72.6%), and K_2_CrO_7_ (75.3%), which is highly toxic. However, the toxicity of the aqueous extract (RT-WAE) was mild at the mortality of 48% of nauplii (Figure 11). In Figure 4, the concentrations of the leaf extracts used range from 0.25 to 4 mg/mL, which is slightly higher than the concentration used for the root extracts due to its less toxicity as confirmed by the preliminary lethal concentration test done. There is no significant difference between the mortality of shrimp larva in the concentrations 0.25–2 mg/mL of the leaf extracts of *R. crispus*. The toxicity of the leaf extract was fully expressed at the concentration of 4 mg/mL. LF-MEE has the highest percentage mortality of 50%, followed by LF-WAE (42.6%) while the LF-ACE has the lowest mortality of 12%. The toxicity of LF-ACE is the least compared to other extracts of the leaf, contrary to the toxicity of RT-ACE, which has the highest toxicity in the root extract (Figures 3 and 4).

#### 3.4.3. Cytotoxicity Activity

**Figure 12:**
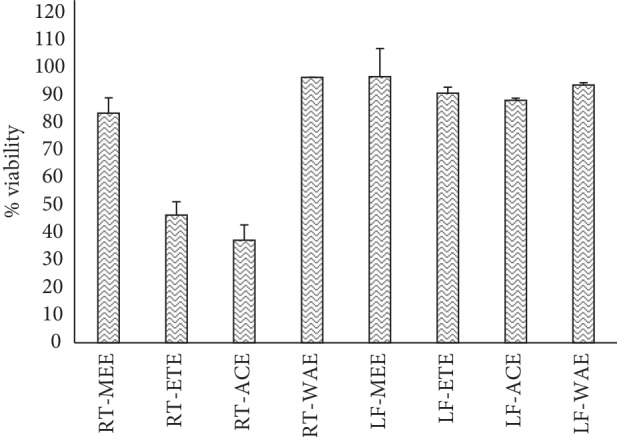
Percentage HeLa cell viability ± SD, obtained after testing on *R. crispus* extracts. RT-MEE: methanol extract of root; RT-ETE: ethanol extract of root; RT-ACE: acetone extract of root; RE-WAE: water extract of root; LF-MEE: methanol extract of leaf; LF-ETE: ethanol extract of leaf; LF-ACE: acetone extract of leaf; LF-WAE: water extract of leaf.

The extracts were tested against HeLa (human cervix adenocarcinoma) cells at a concentration of 50 *μ*g/mL. The result (Figure 12) revealed all extracts are not cytotoxic except RT-ETE (46.55 ± 4.84%) and RT-ACE (37.31 ± 5.69%) which reduce the viability of HeLa cells to below 50%.

## 4. Discussion

The potential of phytomedicine for developing antimicrobial and antiparasitic drugs seems rewarding because most plants that have been tested against microbes and parasites show enormous therapeutic outcome with presumed fewer side effects but these potentials are yet untapped [40, 41]. The genus *Rumex* has been reported and verified scientifically to be effective against a broad range of microorganisms [28, 29, 41, 42] and parasites [43]. The verification of the antimicrobial and antiparasitic potential of *R. crispus* extracts in this research shows that it could be a potential source for new antibacterial, antifungal, antitrypanosomal, and antiplasmodial drugs.

This study revealed extracts of *Rumex crispus* were effective against bacterial growth at different levels of concentrations and there were significant positive correlations between extract concentration and antimicrobial activity through the test for the MIC of bacteria (Table 2) and MIC of fungi (Table 3). In this research, the acetone extract of the root shows a tremendous inhibition power against bacterial growth at the concentration of <1.562 mg/mL compared to other extracts. However, the overall antibacterial potency of the extracts of the root was significantly higher than the extracts of the leaf, which support the previous research done by Orbán-Gyapai et al. [29]. Contrary to the antibacterial potency of the leaf extracts of *R. crispus*, the overall MIC of the extracts shows a higher antifungal potency compared to the root extracts. Furthermore, specific *in vivo* studies are recommended to determine the efficacy of these extracts in the treatment of antimicrobial infections.

The antiplasmodial activity of *R. crispus* extracts was tested and compared with the standard antimalarial drug (chloroquine). Generally, a compound is considered inactive against *Plasmodium* when it shows an IC_50_ > 200 *μ*M, low activity when IC_50_ is 100–200 *μ*M, moderately active when IC_50_ is 20–100 *μ*M, good activity when IC_50_ is 1–20 *μ*M, and excellent or potent antiplasmodial activity when IC_50_ is <1 *μ*M [44]. For natural products or extracts in which the molar mass is difficult to determine, they are categorized as inactive, moderate, good, and excellent antiplasmodials if the IC_50_ values were >5 *μ*g/mL, 1–5 *μ*g/mL, 0.1–1 *μ*g/mL, and <0.1 *μ*g/mL, respectively [45]. RT-ACE and LF-MEE have the highest potency against the *Plasmodium* parasite with an IC_50_ of 13 *μ*g/mL and 15 *μ*g/mL, respectively. However, these two extracts brought about reduction in plasmodium lactate dehydrogenase (pLDH) activity to less than 50% as shown in Figure 1.

The antitrypanosomal activity of the extracts of *R. crispus* was examined using *Trypanosoma brucei* assay. It was found that only RT-ETE reduced the viability of *T.b. brucei* below 50%; hence, RT-ETE was put forward for IC_50_ screening and the IC_50_ value was found to be 9.7 ug/mL. Larayetan et al. [8] confirmed the IC_50_ value of compounds on *Trypanosoma* ≤20 *μ*g/mL are considered good, IC_50_ between 20 and 60 *μ*g/mL are considered moderate, while IC_50_ > 100 *μ*g/mL is termed not active.

The test for toxicity using brine shrimp assay is a viable tool for preliminary evaluation of the toxicity of samples, especially plant extracts [46, 47]. The toxicity of *R. crispus* was evaluated using hatchability of cysts and lethality of the larvae of *Artemia salina* with varying concentrations. In Table 1 and Figures 2–4, 10, shows the lethal concentration (LC) and mean toxicity of the root of *R. crispus* are almost twice as that of the leaf. This could be due to the active phytochemical content of the root which was confirmed to be roughly more than in the leaf [5]. The aqueous extracts also show a mean value of lesser toxicity compared to other solvent extracts except for aqueous extracts of the root that show slightly higher toxicity; hence, the aqueous extracts are presumed to be less toxic when compared to other solvent extracts.

Bastos et al. [48] interpreted the toxicity of brine shrimp in accordance with Meyer toxicity index that LC_50_ values > 1 mg/mL are considered nontoxic and LC_50_ values ≥ 0.5 mg/mL but LC_50_ values <1 mg/mL are considered mild toxic, while LC_50_ values< 0.5 mg/mL are considered highly toxic [48]. Nevertheless, it was found in this study that RT-ETE has the lowest LC_50_ value (LC_50_ 0.275 mg/mL) followed by the LC_50_ value of RT-ACE (LC_50_ 0.363 mg/mL). The response of the larva to LF-ETE (LC_50_ 4.658 mg/mL), LF-MEE (LC_50_ 2.734 mg/mL), LF-WAE (LC_50_ 2.990 mg/mL), and LF-ACE (LC_50_ 1.824 mg/mL) thus reveal the extracts are nontoxic, according to Bastos et al. [48].

To further ascertain the toxicity level of *R. crispus* extracts, cytotoxicity of the extracts was conducted using HeLa (human cervix adenocarcinoma) cells at a concentration of 50 *μ*g/mL. The study shows that RT-MEE, RT-WAE, LF-MEE, LF-ETE, LF-ACE, and LF-WAE did not reduce the percentage viability of HeLa cells below 50%; hence, samples did not cause significant cytotoxic effects, which denote their safety when used as drugs. On the contrary, RT-ETE and RT-ACE reveal some degree of toxicity and reduce the viability of HeLa cells to 46.55 ± 4.84% and 37.31 ± 5.69%, respectively (Figure 12). However, it is worthy of note that there is a correlation between the outcome of brine shrimp lethality assay (BSLA) and cytotoxicity assay using HeLa cells; both assays revealed that RT-ETE and RT-ACE are most toxic among the extracts.

## 5. Conclusion

Based on the results of the research, the extracts of *R*. *crispus* have therapeutic potentials against some microbes (fungal and bacteria) and parasites (*Trypanosoma* and *Plasmodium*). It was confirmed that the antimicrobial activity of the extracts does not dependent on Gram-negative or Gram-positive bacteria and hypha or single-celled-form fungi. The level of toxicity and potency of the extracts correlate with the inhibition of the viability of HeLa cells and *Artemia salina*. The results, therefore, further confirms the use of *Rumex crispus* by traditional healers and recognized the potential of medicinal plants as the prime source of novel compounds in the search of new treatment for African sleeping sickness, malaria, and microbial infections.

## Figures and Tables

**Table 1:** LC_50_ and LC_99_ (mg/mL) and the confidence limits of the dose of solvent extraction of root and leaf of *R. crispus* using brine shrimp lethality assay for an exposure period of 72 h.

Plant extracts	LC_50_ (mg/mL)	LC_99_ (mg/mL)
RT-MEE	0.491 (0.269–0.606)	1.548 (1.051–8.588)
RT-ETE	0.275 (0.174–0.357)	1.196 (0.808–2.997)
RT-ACE	0.363 (0.241–0.440)	0.872 (0.652–2.282)
RT-WAE	0.612 (0.328–0.989)	3.467 (3.126–6.950)
LF-MEE	2.734 (1.535–3.120)	5.678 (5.385–12.120)
LF-ETE	4.658 (2.211–5.399)	17.239 (14.094–22.399)
LF-ACE	1.824 (2.543–3.099)	28.566 (22.204–134.009)
LF-WAE	2.990 (1.309–3.908)	6.854 (4.767–153.999)
K_2_CrO_7_	0.101 (–0.048–0.187)	0.941 (0.706–1.547)

LC: lethal concentration; RT-MEE: methanol extract of root; RT-ETE: ethanol extract of root; RT-ACE: acetone extract of root; RE-WAE: water extract of root; LF-MEE; methanol extract of leaf; LF-ETE: ethanol extract of leaf; LF-ACE: acetone extract of leaf; LF-WAE: water extract of leaf.

**Table 2:** Minimal inhibitory concentration (MIC) of tested bacterial organisms against *R. crispus* extracts.

*R. crispus* extracts (mg/mL)	Gram-negative bacteria				Gram-positive bacteria			
	*K. pneumoniae*	*P. aeruginosa*	*E. coli*	*V. cholerae*	*B. subtilis*	*S. aureus*	*S. pyogenes*	*B. cereus*
RT-MEE	<1.562	6.25	25	<1.562	<1.562	<1.562	<1.562	<1.562
RT-ETE	1.562	1.562	25	<1.562	<1.562	<1.562	<1.562	<1.562
RT-ACE	<1.562	<1.562	<1.562	<1.562	<1.562	<1.562	<1.562	<1.562
RT-WAE	>25	>25	>25	>25	>25	12.5	>25	>25
LF-MEE	<1.562	25	25	<1.562	12.5	12.5	12.5	3.125
LF-ETE	25	>25	>25	25	25	25	25	25
LF-ACE	>25	>25	>25	>25	>25	>25	>25	>25
LF-WAE	>25	>25	>25	>25	>25	>25	>25	>25
Erythromycin (*μ*g/mL)	8	>8	>8	8	>8	2	<0.5	1

RT-MEE: methanol extract of root; RT-ETE: ethanol extract of root; RT-ACE: acetone extract of root; RE-WAE: water extract of root; LF-MEE: methanol extract of leaf; LF-ETE: ethanol extract of leaf; LF-ACE: acetone extract of leaf; LF-WAE: water extract of leaf.

**Table 3:** Minimal inhibitory concentration (MIC) of tested fungal organisms against *R. crispus* extracts and standard drug.

*R. crispus* extracts (mg/mL)	*T. tonsurans*	*T. mucoides*	*P. aurantiogriseum*	*P. chrysogenum*	*C. glabrata*	*C. albicans*
RT-MEE	2.5	2.5	2.5	>10	>10	5
RT-ETE	2.5	<0.625	<0.625	>10	>10	5
RT-ACE	1.25	1.25	1.25	10	>10	2.5
RT-WAE	10	>10	10	>10	>10	>10
LF-MEE	5	2.5	2.5	>10	>10	10
LF-ETE	0.625	0.625	1.25	>10	>10	>10
LF-ACE	0.625	0.625	2.5	>10	>10	5
LF-WAE	1.25	0.625	0.625	>10	>10	>10
Nystatin (*μ*g/mL)	1	1	1	4	32	2

RT-MEE: methanol extract of root; RT-ETE: ethanol extract of root; RT-ACE: acetone extract of root; RE-WAE: water extract of root; LF-MEE: methanol extract of leaf; LF-ETE: ethanol extract of leaf; LF-ACE: acetone extract of leaf; LF-WAE: water extract of leaf.

## Data Availability

On request, the authors can provide the underlying data for the research article.

## References

[1] Amjad M. S., Arshad M., Qureshi R. (2015). Ethnobotanical inventory and folk uses of indigenous plants from Pir Nasoora National Park, Azad Jammu and Kashmir. *Asian Pacific Journal of Tropical Biomedicine*.

[2] Bhalodia N., Shukla V. (2011). Antibacterial and antifungal activities from leaf extracts of Cassia fistula l.: an ethnomedicinal plant. *Journal of Advanced Pharmaceutical Technology & Research*.

[3] Afolayan A. J. (2003). Extracts from the shoots of Arctotis arctotoides inhibit the growth of bacteria and fungi. *Pharmaceutical Biology*.

[4] Selvamohan T., Ramadas V., Kishore S. S. (2012). Antimicrobial activity of selected medicinal plants against some selected human pathogenic bacteria. *Pelagia Research Library Advances in Applied Science Research*.

[5] Idris O. A., Wintola O. A., Afolayan A. J. (2017). Phytochemical and antioxidant activities of Rumex crispus L. in treatment of gastrointestinal helminths in Eastern Cape province, South Africa. *Asian Pacific Journal of Tropical Biomedicine*.

[6] Cragg G. M., Newman D. J. (2005). Biodiversity: a continuing source of novel drug leads. *Pure and Applied Chemistry*.

[7] Murugan K., Aarthi N., Kovendan K. (2015). Mosquitocidal and antiplasmodial activity of Senna occidentalis (Cassiae) and Ocimum basilicum (Lamiaceae) from Maruthamalai hills against Anopheles stephensi and Plasmodium falciparum. *Parasitology Research*.

[8] Larayetan R., Ojemaye M. O., Okoh O. O., Okoh A. I. (2019). Silver nanoparticles mediated by Callistemon citrinus extracts and their antimalaria, antitrypanosoma and antibacterial efficacy. *Journal of Molecular Liquids*.

[9] Srivastava J., Chandra H., Nautiyal A. R., Kalra S. J. S. (2014). Antimicrobial resistance (AMR) and plant-derived antimicrobials (PDAms) as an alternative drug line to control infections. *3 Biotech*.

[10] Dubey D., Rath S., Sahu M. C., Nayak N., Debata N. K., Padhy R. N. (2013). Status of multidrug resistance in tubercle bacillus and phytochemicals for the control. *Journal of Public Health*.

[11] Mishra M. P., Rath S., Swain S. S., Ghosh G., Das D., Padhy R. N. (2017). In vitro antibacterial activity of crude extracts of 9 selected medicinal plants against UTI causing MDR bacteria. *Journal of King Saud University-Science*.

[12] Borchardt J. R., Wyse D. L., Sheaffer C. C. (2008). Journal of medicinal plant research. *Academic Journals*.

[13] Mostafa H. A. M., Elbakry A. A., Eman A. A. (2011). Evaluation of antibacterial and antioxidant activities of different plant parts of Rumex vesicarius L. (polygonaceae). *International Journal of Pharmacy and Pharmaceutical Sciences*.

[14] Martinez-Correa H. A., Bitencourt R. G., Kayano A. C. A. V., Magalhães P. M., Costa F. T. M., Cabral F. A. (2017). Integrated extraction process to obtain bioactive extracts of Artemisia annua L. leaves using supercritical CO_2_, ethanol and water. *Industrial Crops and Products*.

[15] Nardella F., Gallé J.-B., Bourjot M., Weniger B., Vonthron-Sénécheau C. (2018). Antileishmanial and antitrypanosomal activities of flavonoids. *Sustainable Development and Biodiversity*.

[16] Savoia D. (2012). Plant-derived antimicrobial compounds: alternatives to antibiotics. *Future Microbiology*.

[17] Kuria M. ., Njenga P. K., Ngumi V. . (2012). Ethnobotanical studies of Strychnos henningsii in five (Gilg.) natural habitats in Kenya. *International Journal of Medicinal Plants Research*.

[18] Idris O. A., Wintola O. A., Afolayan A. J. (2019). Helminthiases; prevalence, transmission, host-parasite interactions, resistance to common synthetic drugs and treatment. *Heliyon*.

[19] Githiori J. B., Athanasiadou S., Thamsborg S. M. (2006). Use of plants in novel approaches for control of gastrointestinal helminths in livestock with emphasis on small ruminants. *Veterinary Parasitology*.

[20] Bello T. H., Idris O. A. (2018). The effect of antioxidant (gallic acid) on the testes of lead acetate induced Wistar rat. *Toxicology and Environmental Health Sciences*.

[21] Idris O., Wintola O., Afolayan A. (2019). Comparison of the proximate composition, vitamins (ascorbic acid, *α*-tocopherol and retinol), anti-nutrients (phytate and oxalate) and the GC-MS analysis of the essential oil of the root and leaf of Rumex crispus L.. *Plants*.

[22] Mayorga P., Pérez K. R., Cruz S. M., Cáceres A. (2010). Comparison of bioassays using the anostracan crustaceans Artemia salina and Thamnocephalus platyurus for plant extract toxicity screening. *Revista Brasileira de Farmacognosia*.

[23] Hamidi M. R., Jovanova B., Kadifkova Panovska T. (2014). Toxicological evaluation of the plant products using brine shrimp (Artemia salina L.) model. *Macedonian Pharmaceutical Bulletin*.

[24] Veni T., Pushpanathan T. (2014). Comparison of the Artemia salina and Artemia fransiscana bioassays for toxicity of Indian medicinal plants. *Journal of Coastal Life Medicine*.

[25] Parra A. L., Yhebra R. S., Sardiñas I. G., Buela L. I. (2001). Comparative study of the assay of Artemia salina L. and the estimate of the medium lethal dose (LD50 value) in mice, to determine oral acute toxicity of plant extracts. *Phytomedicine*.

[26] Vasas A., Orbán-Gyapai O., Hohmann J. (2015). The genus Rumex: review of traditional uses, phytochemistry and pharmacology. *Journal of Ethnopharmacology*.

[27] Coruh I., Gormez A., Ercisli S., Sengul M. (2008). Total phenolic content, antioxidant, and antibacterial activity of Rumex crispus grown wild in Turkey. *Pharmaceutical Biology*.

[28] Wegiera M., Kosikowska U., Malm A., Smolarz H. (2011). Antimicrobial activity of the extracts from fruits of Rumex L. species. *Open Life Sciences*.

[29] Orbán-Gyapai O., Liktor-Busa E., Kúsz N. (2017). Antibacterial screening of Rumex species native to the Carpathian Basin and bioactivity-guided isolation of compounds from Rumex aquaticus. *Fitoterapia*.

[30] Worku N., Mossie A., Stich A. (2013). Evaluation of the in vitro efficacy of Artemisia annua , Rumex abyssinicus , and Catha edulis Forsk extracts in cancer and Trypanosoma brucei cells. *ISRN Biochemistry*.

[31] Lee K. H., Rhee K.-H. (2013). Antimalarial activity of nepodin isolated from Rumex crispus. *Archives of Pharmacal Research*.

[32] Andrews J. M. (2001). Determination of minimum inhibitory concentrations. *Journal of Antimicrobial Chemotherapy*.

[33] Mostafa A. A., Al-Askar A. A., Almaary K. S., Dawoud T. M., Sholkamy E. N., Bakri M. M. (2018). Antimicrobial activity of some plant extracts against bacterial strains causing food poisoning diseases. *Saudi Journal of Biological Sciences*.

[34] Lass-Flörl C., Cuenca-Estrella M., Denning D. W., Rodriguez-Tudela J. L. (2006). Antifungal susceptibility testing in Aspergillusspp. according to EUCAST methodology. *Medical Mycology*.

[35] Jain S., Jacob M., Walker L., Tekwani B. (2016). Screening North American plant extracts in vitro against Trypanosoma brucei for discovery of new antitrypanosomal drug leads. *BMC Complementary and Alternative Medicine*.

[36] Otunola G. A., Unuofin J. O., Afolayan A. J. (2017). Toxicity evaluation of Vernonia mespilifolia less (a South Africa medicinal plant) using brine shrimp. *Journal of Pharmacology and Toxicology*.

[37] Abosede W. O., Sunday A., Jide A. A. (2015). Toxicological investigations of Aloe ferox Mill. extracts using brine shrimp (Artemia salina L.) assay. *Pakistan Journal of Pharmaceutical Sciences*.

[38] Agrafioti P., Athanassiou C. G., Vassilakos T. N., Vlontzos G., Arthur F. H. (2015). Using a lethality index to assess susceptibility of Tribolium confusum and Oryzaephilus surinamensis to insecticides. *PLoS One*.

[39] Milhem M. M., Al-Hiyasat A. S., Darmani H. (2008). Toxicity testing of restorative dental materials using brine shrimp larvae (Artemia salina). *Journal of Applied Oral Science*.

[40] Wink M. (2012). Medicinal plants: a source of anti-parasitic secondary metabolites. *Molecules*.

[41] Maryam M. S., Parvin S., Mahboobeh M. (2013). Evaluation of antibacterial activity of extract of Rumex alveollatus leaf against Staphylococcus aureus and Pseudomonas aeruginosa. *Zahedan Journal of Research in Medical Sciences*.

[42] Yadav S., Kumar S., Jain P. (2011). Antimicrobial activity of different extracts of roots of Rumex nepalensis Spreng. *Indian Journal of Natural Products and Resources*.

[43] Cock I. E., Selesho M. I., Van Vuuren S. F. (2018). A review of the traditional use of Southern African medicinal plants for the treatment of selected parasite infections affecting humans. *Journal of Ethnopharmacology*.

[44] Nogueira C. R., Lopes L. M. X. (2011). Antiplasmodial natural products. *Molecules*.

[45] Lemma M. T., Ahmed A. M., Elhady M. T. (2017). Medicinal plants for in vitro antiplasmodial activities: a systematic review of literature. *Parasitology International*.

[46] Wintola O., Afolayan A. (2015). An inventory of indigenous plants used as anthelmintics in Amathole district municipality of the Eastern Cape province, South Africa. *African Journal of Traditional, Complementary and Alternative Medicines*.

[47] Afolayan A. J., Ohikhena F. U., Wintola O. A. (2016). Toxicity assessment of different solvent extracts of the medicinal plant, Phragmanthera capitata (Sprengel) Balle on brine shrimp (Artemia salina). *International Journal of Pharmacology*.

[48] Bastos M., Lima M., Conserva L. M., Andrade V. S., Rocha E. M., Lemos R. P. (2009). Studies on the antimicrobial activity and brine shrimp toxicity of Zeyheria tuberculosa (Vell.) Bur. (Bignoniaceae) extracts and their main constituents. *Annals of Clinical Microbiology and Antimicrobials*.

